# Growth and non-structural carbohydrates response patterns of *Eucommia ulmoides* under salt and drought stress

**DOI:** 10.3389/fpls.2024.1436152

**Published:** 2024-07-18

**Authors:** Xuejie Zhang, Hao Qin, Zhenchao Kan, Dan Liu, Bingxin Wang, Shoujin Fan, Peipei Jiang

**Affiliations:** ^1^ Key Lab of Plant Stress Research, College of Life Sciences, Shandong Normal University, Ji’nan, China; ^2^ Dongying Key Laboratory of Salt Tolerance Mechanism and Application of Halophytes, Dongying Institute, Shandong Normal University, Dongying, China; ^3^ Shandong Provincial Center of Forest and Grass Germplasm Resources, Ji’nan, China; ^4^ Dalin Eucommia planting company of Gaomi County, Weifang, China

**Keywords:** droughts, leaf mass area, relative growth rate, non-structural carbohydrates, root/shoot ratio, salt stress

## Abstract

**Introduction:**

Salinity and droughts are severe abiotic stress factors that limit plant growth and development. However, the differences and similarities of non-structural carbohydrates (NSCs) responses patterns of trees under the two stress conditions remain unclear.

**Methods:**

We determined and compared the growth, physiology, and NSCs response patterns and tested the relationships between growth and NSCs concentrations (or pool size) of *Eucommia ulmoides* seedlings planted in field under drought and salt stress with different intensities and durations.

**Results and discussion:**

We found that drought and salt stress can inhibit the growth of *E. ulmoides*, and *E. ulmoides* tended to enhance its stress resistance by increasing proline concentration and leaf thickness or density but decreasing investment in belowground biomass in short-term stress. During short-term drought and salt stress, the aboveground organs showed different NSCs response characteristics, while belowground organs showed similar change characteristics: the starch (ST) and NSCs concentrations in the coarse roots decreased, while the ST and soluble sugar (SS) concentrations in the fine roots increased to enhance stress resistance and maintain water absorption function. As salt and drought stress prolonged, the belowground organs represented different NSCs response patterns: the concentrations of ST and SS in fine roots decreased as salt stress prolonged; while ST in fine roots could still be converted into SS to maintain water absorption as drought prolonged, resulting in an increase of SS and a decrease of ST. Significant positive relationships were found between growth and the SS and total NSCs concentrations in leaves and branches, however, no significant correlations were found between growth and below-ground organs. Moreover, relationships between growth and NSCs pool size across organs could be contrast.

**Conclusion:**

Our results provide important insights into the mechanisms of carbon balance and carbon starvation and the relationship between tree growth and carbon storage under stress, which were of great significance in guiding for the management of artificial forest ecosystem under the context of global change.

## 1 Introduction

Drought would become more frequent, intense, and long-lasting under climate change, and has become a major factor affecting global forest mortality (Carnicer et al., 2011; Trenberth et al., 2014; Cramer et al., 2018). Salinity is another major abiotic stress limiting plant growth and development (Aragüés et al., 2015; Wang et al., 2021). Increasing irrigation under climate change could lead to further salinization, since the dissolved salts in irrigation water can be transported back to surface through water uptake and evaporation (Polle and Chen, 2015; Xu et al., 2022). Salinity, similarly to droughts, represents physiological dryness and results in osmotic stress in plants (Munns, 2002; Li et al., 2016; Wang et al., 2021). Also, salt stress destroys the ion balance in plants, and its harm will be more severe than low osmotic stress alone (Chaves et al., 2009; Zhang et al., 2010). Most researches focused on plant response to droughts or salinity alone, however, researches conducted to compare plants’ response differences under the two stress conditions simultaneously are still lacking.

Non-structural carbohydrates (NSCs), primarily composed of soluble sugar (SS) and starch (ST), are necessary energy sources for the growth and metabolism in plants, and are also major contributors to plant structure construction (Chapin et al., 1990; Hoch et al., 2003). A lot of researches showed that NSCs support multiple functions in metabolism, osmoregulation and defense and therefore play critical roles in stress resistance and resilience (Bartlett et al., 2012; Dietze et al., 2014; Piper et al., 2022; Blumstein et al., 2023). Moreover, SS can not only repair the xylem embolism to maintain efficient water transport (Secchi and Zwieniecki, 2011; Trifilò et al., 2019), but also help detoxify by serving as a chelating agent to capture Na^+^ within starch granules (Kanai et al., 2007). Moreover, the allocation of SS and ST in trees is dynamic and a mutual conversion relationship was found between them under certain conditions (Latt et al., 2001; Gibon et al., 2009; Jiao et al., 2020), and thus NSCs can act as an index to evaluate the level of available substances in plants and the balance between carbon sources and sinks (Iglesias et al., 2002; Kannenberg et al., 2018; Furze et al., 2019). Therefore, it is important to study the changes of NSCs and its composition and distribution patterns in trees under stress conditions to reveal the ecological adaptation strategies and stress resistance mechanisms of trees to changing environments.

A lot of studies found that plants would increase the concentrations of NSCs under environmental stress (Anderegg et al., 2012; Piper et al., 2017; Zhou et al., 2021). This may be because higher concentrations of NSCs can better meet plants’ osmotic demands under environmental stress (Dietze et al., 2014; Guo et al., 2020; Sapes et al., 2021). Also, trees with higher concentrations of NSCs could maintain higher stem water potential and therefore could survive longer under droughts (O’Brien et al., 2014). Moreover, it is assumed that the higher demands of NSCs for sapling survival might be evolutionarily more favorable in the event of recurrent cavitation risk (Sala et al., 2012; Piper et al., 2022). However, some studies showed that NSCs concentrations remained unchanged (Gruber et al., 2012; Dickman et al., 2019) or declined under droughts (Galiano et al., 2012; Adams et al., 2013; Dickman et al., 2015). The reason for these contrast conclusions might because differences in the intensity and duration of droughts (McDowell, 2011; Rosas et al., 2013; He et al., 2020). Second, this might also be related to plant characteristics, e.g. plant size and water use strategies (Mitchell et al., 2013; Garcia-Forner et al., 2017; Zhang et al., 2020). In addition, different plant organs also have different response strategies to environmental stress. For example, studies have shown that droughts significantly decreased the concentration of root NSCs, while the concentration of NSCs in the aboveground part remained unchanged (Hartmann et al., 2013; Li W. et al., 2018). In contrast, in some tree species, although the distribution of newly synthesized carbohydrates to the belowground part was significantly decreased, the storage of NSCs in belowground part increased (Galvez et al., 2011; Hagedorn et al., 2016). Most of these researches are focused on drought, shade or low temperature stress experiments, however, the allocation mechanism of NSCs under salt stress is seriously insufficient. Therefore, it is desirable to research the distribution patterns of NSCs and its composition across plant organs under salt stress conditions of different intensities and duration.

Allocation of NSCs to storage allows plants to maintain a carbon pool to cope with stress, however, the priority allocation to storage could compete with growth and thus created a trade-off between them (McDowell, 2011; Wiley and Helliker, 2012; Stefaniak et al., 2024). This has been confirmed by the negative correlation between growth and NSCs storage in many studies (Silpi et al., 2007; Chantuma et al., 2009; Genet et al., 2010). However, others studies showed the opposite results (Poorter and Kitajima, 2007; Imaji and Seiwa, 2010). Also, a recent study showed radial growth was decoupled with NSCs concentrations and pool sizes in broadleaf temperate tree species (Piper, 2020). These results indicate that there are still many uncertainties in the relationship between growth and storage, and further studies are needed.

*Eucommia ulmoides* Oliver (*E. ulmoides*) is the sole species of the genus *Eucommia* and is a precious medicinal and economic tree species indigenous to China (He et al., 2014; Zhu and Sun, 2018). *E. ulmoides* contains a specific white filamentous material, gutta-percha gum, which is extensively used in various fields of life (Liu et al., 2022; Zhao et al., 2023). Under the global climate change situation, the growth and development of trees are increasingly threatened by drought and salt stress (Polle and Chen, 2015; Choat et al., 2018; McDowell et al., 2020; Yu et al., 2023). Recent researches on the response of *E. ulmoides* to salt and drought stress mainly focus on the morphology, physiology and molecular biology (Wang et al., 2018; Li et al., 2019; Zuo et al., 2022), however, its response patterns of NSCs and its composition under stress is still not clear. As well as we known, no study has been conducted to compare the similarities and differences of its NSCs response patterns under both salt and drought stress simultaneously. Therefore, we measured the relative growth rate, leaf mass area, root/shoot ratio, leaf proline and chlorophyll concentrations, and the NSCs and its compositions concentrations and pool size across different organs of *E. ulmoides* seedlings planted in the field under different drought and salt stress intensities and durations. We aimed to: (i) clarify the variation patterns of growth and physiological indicators in *E. ulmoides* under salt and drought stress; (ii) elucidate the allocation patterns of NSCs and its compositions across different organs in *E. ulmoides* under salt and drought stress; (iii) compare the similarities and differences of morphological and physiological indicators and NSCs response patterns under salt and drought stress; (iv) determine the relationship between the relative growth rate and NSCs concentration and pool size under stress.

## 2 Materials and methods

### 2.1 Site description

This research was carried out at the south side of Shandong Normal University at the foot of Shuanglong Mountain (36°31′N, 116°49′E), located in the Shandong Province of eastern China. Affected by a warm temperate continental monsoon, the study area has four distinct seasons. The annual average rainfall is 623.1 mm, and the annual average temperature is 13.8°C. The main soil types are yellow brown soil and brown soil.

### 2.2 Experimental design

In March 2022, 18 quadrats (2 ×0.75 m) were set in our experimental plot. The horizontal interval between these quadrats was 1 m, and the longitudinal interval is 0.5 m. Each quadrate was excavated to a depth of 0.75 m and surrounded with a 12 mm thick plastic cloth (including the sides and bottom), and then the excavated soil was mixed and filled back. Annual seedlings of *E. ulmoides* with relatively consistent basal diameter and height were selected in Dalin Forest Farm of Weifang City in Shandong Province (36°18′N, 119°37′E), and then transplanted in our experimental plot. Twelve saplings were evenly planted in each quadrat. Then, water these seedlings normally for three months to ensure their healthy growth and development. In June 2022, three quadrats were selected for natural drought stress: the experiment started by stopping watering the plants until harvest. Meanwhile, five salt concentration gradients were set: 0 mM (control check, CK), 50 mM, 100 mM, 150 mM, 200 mM. Three quadrats were set for each salt concentration treatment. Each salt concentration treatment was irrigated with the same volume of corresponding concentration of salt solution per irrigation, and the control check group was irrigated with the same volume of tap water. Then irrigate the field according to the soil water content until harvest. Prepare a rain shelter for each quadrat, remove it on sunny days to keep the seedlings in natural light, and build it on rainy days to avoid the potential effect of precipitation on our experiments. By observing phenotype changes in these seedlings, we carried out two harvest sampling on day 30 and day 60 after drought (D1 and D2 period) and salt (S1 and S2 period) treatments. Each indicator was measured for at least five individuals in each treatment.

### 2.2 Measurement of relative growth rate

We determined the base diameter with vernier caliper at the beginning of the drought and salt stress treatment and at two sampling time. Relative growth rate (RGR) of the *E. ulmoides* seedlings was calculated as:


$$\text{RGR}=\frac{\ln\left(BD_{2}/BD_{1}\right)}{T_{2}-T_{1}}$$

where *BD*_1_ and *BD*_2_ represent base diameters determined at time *T*_1_ and *T*_2_, respectively (Li Y. et al., 2018).

### 2.3 Measurements of leaf mass area, and the concentrations of proline and chlorophyll

In July and August 2022, 15 healthy leaves were collected per treatment to determine the leaf mass area (LMA). After scanning the leaves, the leaf areas were obtained by ImageJ software (1.46r, Bethesda, USA). Then, dry these leaves at 65°C in an oven to constant and weighed. LMA was then calculated as the ratio of leaf dry weight to its area. For biochemical parameters measurement, enough fresh leaves of each treatment were collected in July and August 2022, respectively. The proline concentration was determined by sulfosalicylic acid method (Yang et al., 1999). Pigments in leaves were extracted by using 95% (v/v) ethanol and determined by an ultraviolet spectrophotometer (UV-330, Cambridge, UK). See Zhu et al. (2021) for the details.

### 2.4 Measurement of the root to shoot ratio and non-structural carbohydrates concentration

In July and August 2022, seedlings of each treatment were harvested. Each individual was divided into five parts: leaf, branch, stem, coarse root, and fine root (< 2 mm). All plant samples were oven-dried at 105°C for 1 h to stop all enzymatic activity, then dry them at 65°C to constant. The root to shoot (R/S) ratio was calculated as the weight of the belowground part (coarse root and fine root) divided by that the weight of the aboveground part (leaf, branch, and stem). Then, plant samples were ground into a fine powder for NSCs analysis. SS in plant samples were extracted three times by centrifugation using 80% v/v ethanol. Following sugar extraction, the remaining tissue was firstly solubilized and then hydrolyzed to glucose using enzymes, and then the supernatants were used to assay the starch concentration. SS and ST determinations were measured at 620 nm by an ultraviolet spectrophotometer (UV-330, Cambridge, UK). Total NSCs concentration was defined as the total concentrations of SS and ST on the percentage of dry matter basis.

### 2.5 Data analysis

The SS/ST/NSCs pool size of each organ was calculated as the product of SS/ST/NSCs concentration of each organ and its dry mass, and the SS/ST/NSCs pool size at the whole plant level was calculated as the sum of pool size across organs. One-way ANOVA was conducted to determine the differences in RGR, LMA, R/S ratio, chlorophyll and proline concentrations, and the concentrations of total NSCs and its component across plant organs amongst different stress treatments in SPSS (v.19.0, Chicago, USA). The independent sample t-test was conducted to determine the differences in above indicators between the same stress treatment in SPSS. The significance of above tests was at the 0.05 level. Pearson’s correlation was used to determine the relationships between RGR and the concentrations of SS/ST/NSCs concentration and their pool size across organs and the whole plant level in SPSS. During the Pearson correlation, highly significant and moderately significant were defined as *P*< 0.05 and 0.10, respectively.

## 3 Results

### 3.1 Response patterns of growth and physiological indicators under drought and salt stress

**Figure 1:**
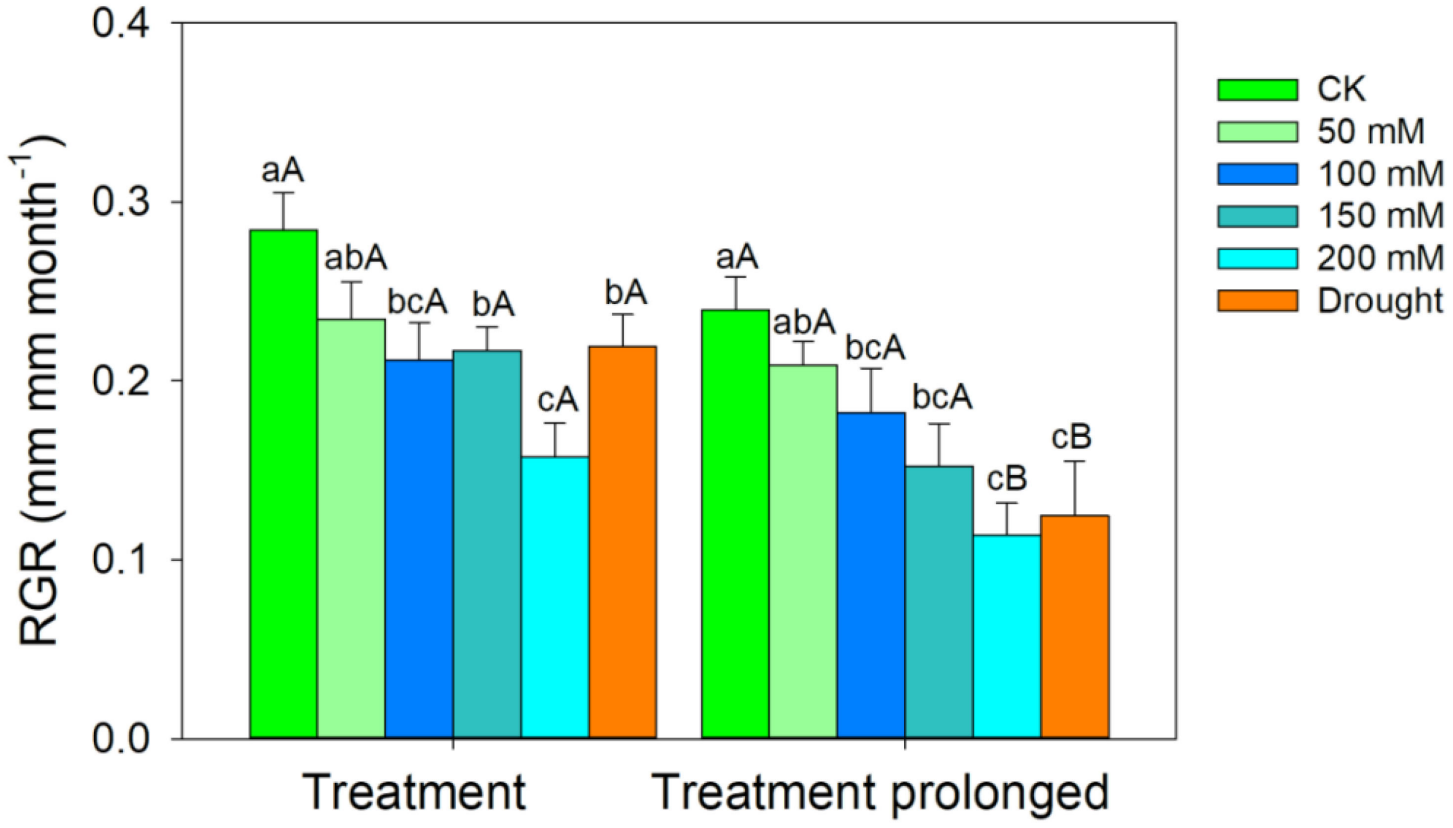
Variation patterns of the relative growth rate (RGR) in response to salt and drought stress. Different lowercase letters indicate significant differences amongst different treatments (*P*<0.05). Different uppercase letters indicate significant differences between the early and late stage of different treatments (*P*<0.05).

**Figure 2:**
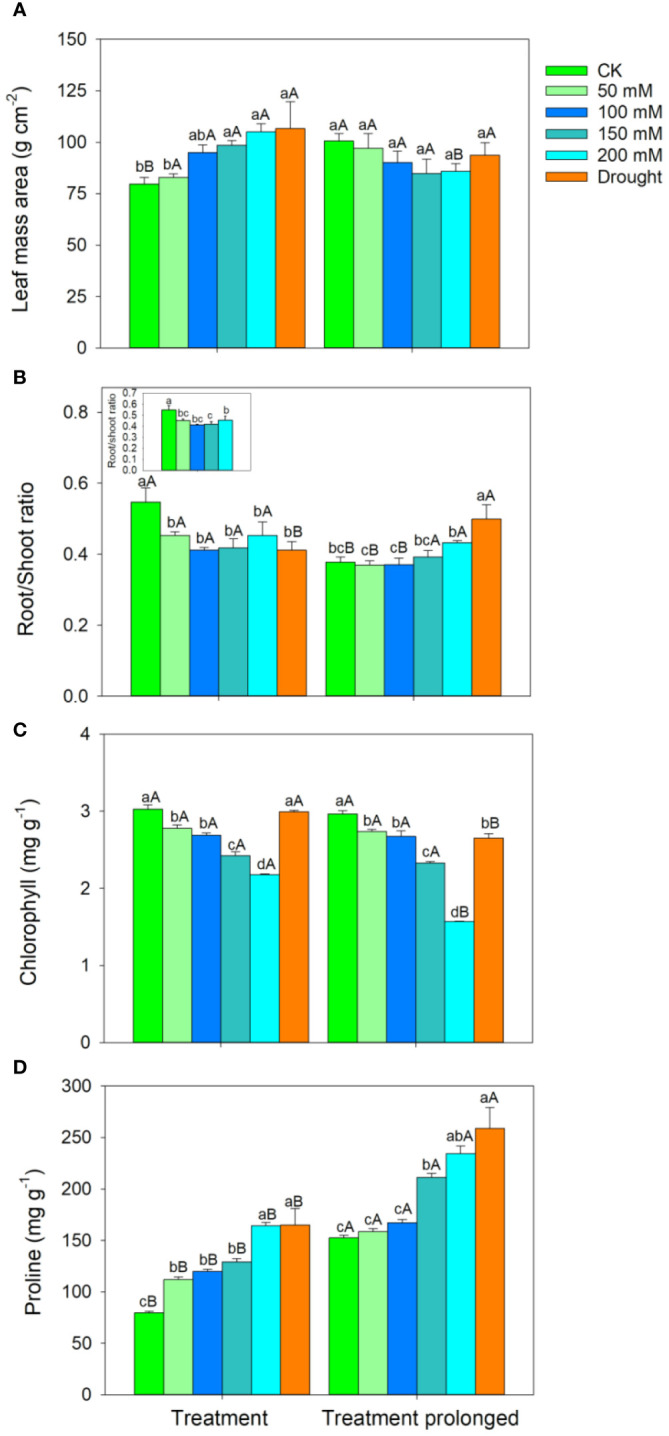
Variation patterns of leaf mass area **(A)**, root to shoot ratio **(B)**, chlorophyll concentration **(C)**, proline concentration **(D)** in response to salt and drought stress. The sub-figure showed the variation pattern of the root to shoot ratio in the early stage of salt stress. Different lowercase letters indicate significant differences amongst different treatments (P<0.05). Different uppercase letters indicate significant differences between the early and late stage of differenttreatments (P<0.05).

The RGR decreased with the increase of stress intensities and durations (**Figure 1**). The LMA increased with salt concentration in the early period of salt stress (S1 period), but it remained unchanged in the late period of salt stress (S2 period) (**Figure 2A**). The R/S ratio decreased with salt concentration in the S1 period, but it increased with salt concentration in the S2 period (**Figure 2B**). Similarly, the LMA increased in the early stage of drought stress (D1 period), but no significant differences were found between that in the late period of drought (D2 period) and CK (**Figure 2A**). The R/S ratio decreased in the D1 period, but it increased as drought stress prolonged (**Figure 2B**).

In both the S1 and S2 periods, the chlorophyll concentration decreased, while the proline concentration increased with the increase of salt concentration (**Figures 2C, D**). As salt stress prolonged, no significant differences were found in chlorophyll concentration between the S1 and S2 period except for in salt concentration of 200 mM (**Figure 2C**), while the proline concentration was higher in the S2 period than that in the S1 period in each salt concentration (**Figure 2D**). Compared with those in CK, there was no significant change in chlorophyll concentration in the D1 period, but chlorophyll concentration decreased significantly in D2 period (**Figure 2C**). Compared with those in CK, the proline concentration increased significantly in both the D1 and D2 period (**Figure 2D**). As drought stress prolonged, the chlorophyll concentration decreased and the proline concentration increased (**Figures 2C, D**).

### 3.2 Response patterns of non-structural carbohydrates under drought and salt stress

**Figure 3:**
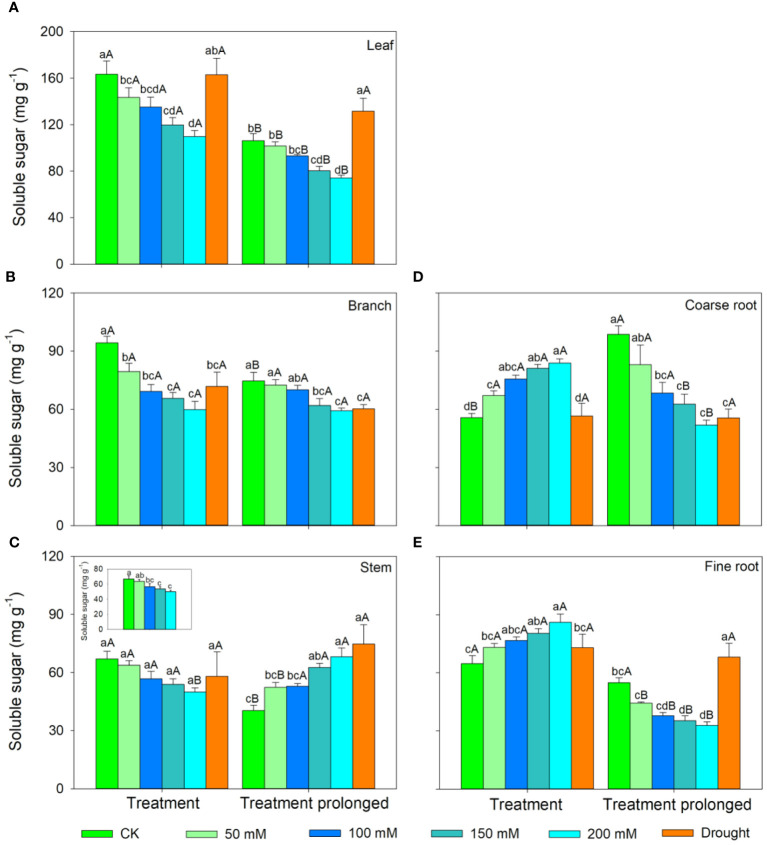
Variation patterns of soluble sugar concentration across different organs (**A**, leaf; **B**, branch; **C**, stem; **D**, coarse root; **E**, fine root) in response to salt and drought stress. The sub-figure showed the variation pattern of soluble sugar concentration in stem in the early stage of salt stress. Different lowercase letters indicate significant differences amongst different treatments (P<0.05). Different uppercase letters indicate significant differences between the early and late stage of different treatments (P<0.05).

**Figure 4:**
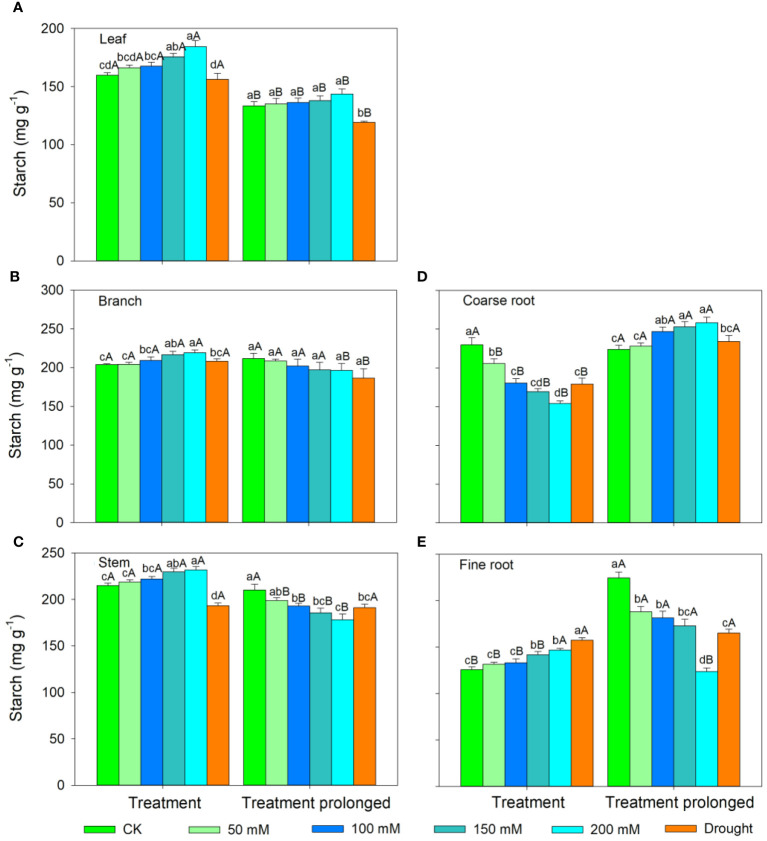
Variation patterns of starch concentration across different organs (**A**, leaf; **B**, branch; **C**, stem; **D**, coarse root; **E**, fine root) in response to salt and drought stress. Different lowercase letters indicate significant differences amongst different treatments (P<0.05). Different uppercase letters indicate significant differences between the early and late stage of different treatments (P<0.05).

**Figure 5:**
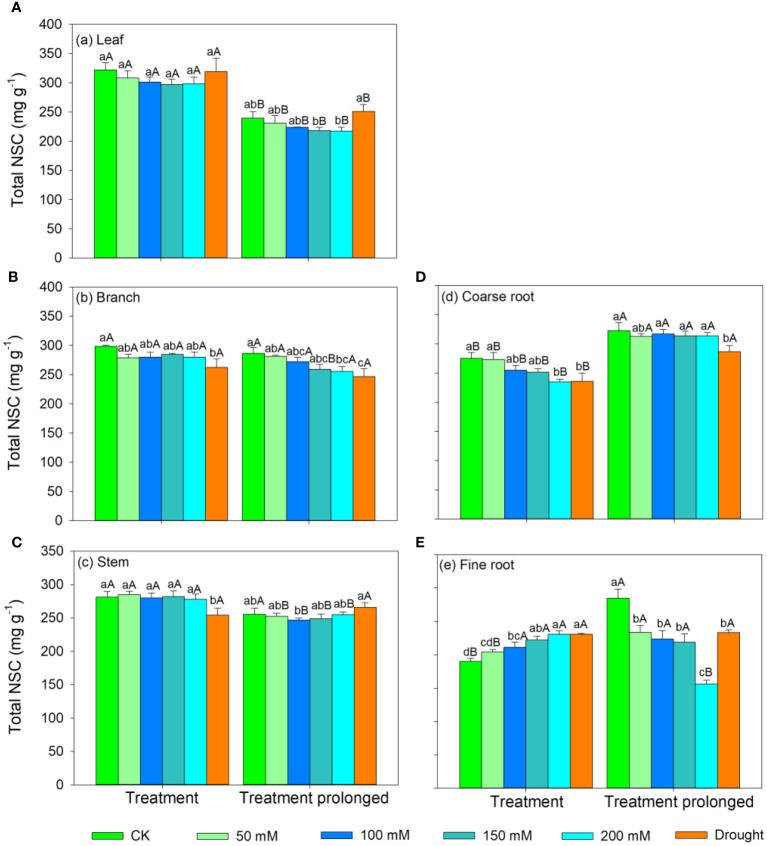
Variation patterns of the total NSCs concentration across different organs (**A**, leaf; **B**, branch; **C**, stem; **D**, coarse root; **E**, fine root) in response to salt and drought stress. Different lowercase letters indicate significant differences amongst different treatments (P<0.05). Different uppercase letters indicate significant differences between the early and late stage of different treatments (P<0.05).

In the S1 period, the SS concentration in leaves, branches, and stem decreased with salt concentration, while the ST concentration showed an increasing trend (**Figures 3**, **4**). Therefore, the concentration of the total NSCs remained unchanged with salt concentration (**Figure 5**). The SS concentration in both coarse and fine roots increased with salt concentration (**Figure 3**). The ST and NSCs concentrations in coarse root decreased, while those in fine root increased with salt concentration (**Figures 4**, **5**). In the S2 period, the SS concentration in leaves decreased, the ST concentration remained unchanged, and total NSCs concentration slightly decreased (though not significant) with salt concentration (**Figures 3**–**5**). The SS and total NSCs concentrations in branches decreased (**Figures 3**, **5**), while ST concentration remained unchanged with salt concentration (**Figure 4**). The SS concentration in stem increased, while the ST concentration decreased with salt concentration, and thus resulting in no significant change in total NSCs (**Figures 3**–**5**). The SS concentration in coarse root decreased, the ST concentration increased with salt concentration, and therefore the concentration of total NSCs remained unchanged (**Figures 3**–**5**). The SS, ST and total NSCs concentrations in fine root all decreased with salt concentration (**Figures 3**–**5**).

In the D1 period, the SS concentration in branch decreased, while it showed no significant changes in other organs compared with those in CK (**Figure 3**). The ST concentration in leaves and branches remained stable, the ST concentration in stems and coarse roots decreased, and the ST concentration in fine roots increased compared with those in CK (**Figure 4**). As a result, leaf NSCs concentration remained unchanged, those in branches, stems and coarse roots decreased, and that in fine roots increased (**Figure 5**). In the D2 period, the SS concentration in leaves, stems and fine roots increased, while the ST concentration decreased compared with those in CK (**Figures 3**, **4**). The SS concentration in branches and coarse roots decreased, while the ST concentration remained unchanged compared with those in CK (**Figures 3**, **4**). The total NSCs concentration in leaves and stems remained unchanged, while those in branches, coarse roots and fine roots decreased (**Figure 5**).

### 3.3 Relationships between relative growth rate and non-structural carbohydrates concentration and pool size

**Figure 6:**
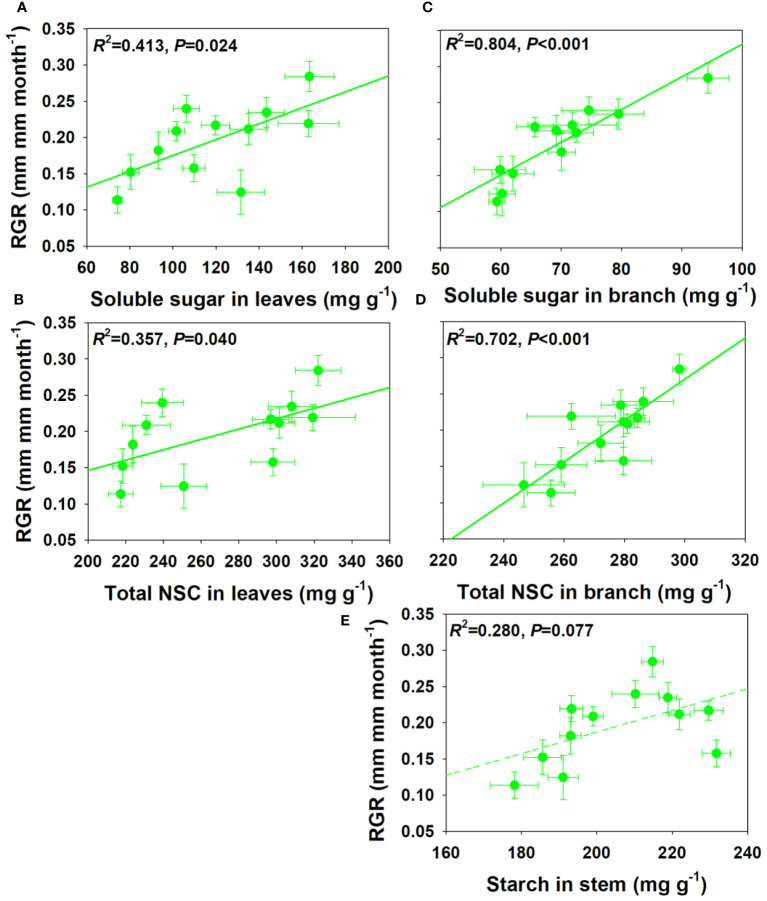
Relationships between relative growth rate (RGR) and soluble sugar and total NSCs concentrations in leaves **(A, B)** and branch **(C, D)**, and starch in stem under salt and drought stress **(E)**.

**Figure 7:**
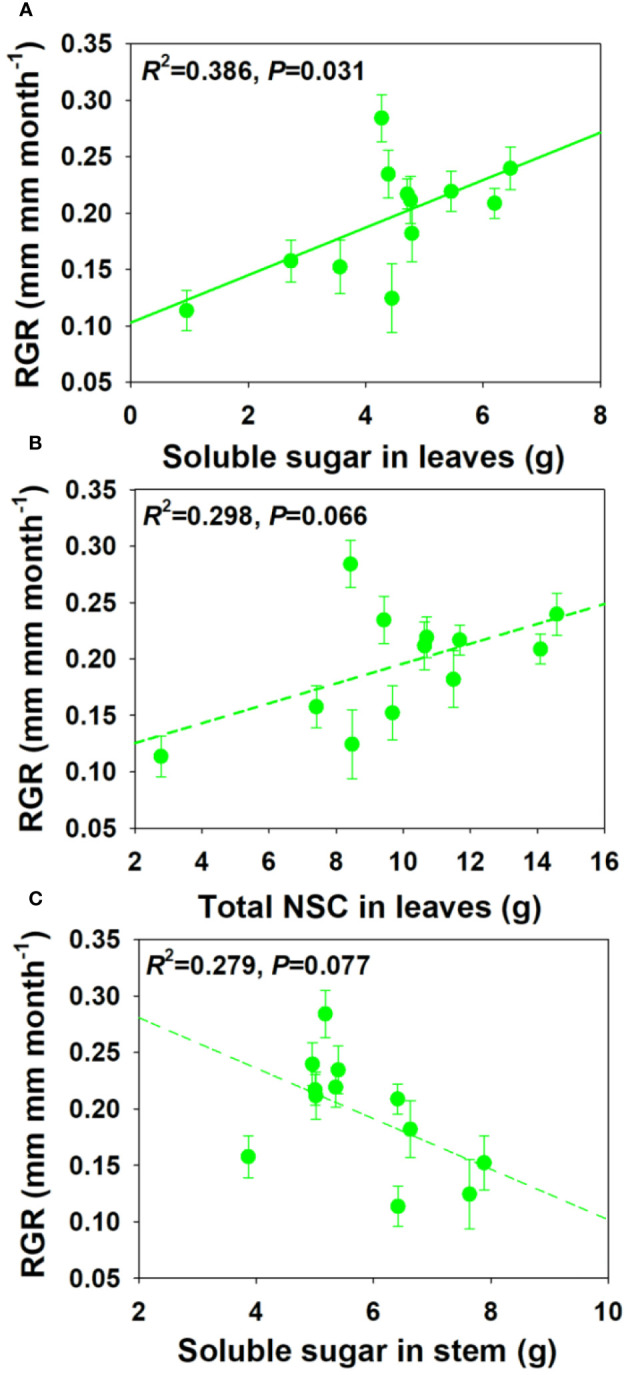
Relationships between relative growth rate (RGR) and soluble sugar and total NSCs pool size in leaves **(A, B)** and soluble sugar pool size in stem under salt and drought stress **(C)**.

For NSCs and its composition concentration, RGR showed significant positive correlations with SS and total NSCs concentrations in leaves and branch, and a moderately positive correlation with ST concentration in stem under drought and salt stress (**Figure 6**). For NSCs and its composition pool size, RGR showed significant and moderately significant correlations with SS and total NSCs pool size in leaves, but a moderately negative correlation with SS pool size in stem under drought and salt stress (**Figure 7**). The relationships between RGR and ST/SS/NSCs concentration and pool size under salt stress were similar with those under drought and salt stress (**Supplementary Figures S1**, **S2**). However, no significant correlations were found between growth and ST/SS/NSCs concentration and pool size of below-ground organs and the whole plant level (**Supplementary Tables S1**-**S2**).

## 4 Discussion

### 4.1 Comparisons of growth and physiological indicators response patterns under drought and salt stress

The R/S ratio can reflect the adjustment strategy of biomass in the above- and belowground parts of plants under stress conditions, reflecting the “functional balance” in resource allocation, which helps plants maintain optimal growth status under environmental changes (Moser et al., 2015; Bacher et al., 2022). Past studies showed that some plants will decrease the proportion of root biomass, thereby reducing the absorption of salt by roots, while other plants reduce water loss by decreasing the accumulation of aboveground biomass to maintain water and enhance their survival ability (Salter et al., 2006). We found that the R/S ratio decreased with salt concentration in the S1 period, while it increased with salt concentration in the S2 period. This indicated that the allocations of above- and belowground biomass in *E. ulmoides* showed a transition between the above two strategies as salt stress prolonged. This may be because the root system underwent ion toxicity and withered under the action of salt solution during the S1 period, resulting in the decrease of root biomass and thus reducing the salt absorption by roots (Jacqueline et al., 2006; Fan et al., 2011). As salt stress prolonged, the leaves of *E. ulmoides* fell off with the increase of salt concentration, resulting in an increase in R/S ratio (Cimato et al., 2010; Luo et al., 2011). Since fine root biomass could decrease due to the decrease of root elongation and increase of root cavitation in short-term droughts (Joslin et al., 2000; Nordborg and Welander, 2001), the R/S ratio in our study decreased in the D1 period. Differences in LMA between species can reflect potential variations in leaf anatomic traits, such as thickness and density of cuticle and mesophyll cell, as well as stability of cellular structure (John et al., 2017). Meanwhile, the LMA of *E. ulmoides* increased in the D1 period. These evidences collectively indicated that *E. ulmoides* tended to enhance its stress resistance by increasing leaf thickness or density and decreasing investment in belowground biomass in short-term drought (Fernández et al., 2002; Liu et al., 2020; Sancho-Knapik et al., 2021). With the continuous progress of droughts, LMA maintained at a high level to facilitate the resistance to drought, and the roots tended to dig into deeper soil to more effectively absorb water and thus leading to an increase of the R/S ratio. Similarly, several studies suggested that woody species tended to increase their belowground biomass allocation to increase deep soil water utilization over the long-term droughts (Kir et al., 2013; Aaltonen et al., 2017).

The chlorophyll concentration is directly correlated with the photosynthesis rate of plants, and its changes can to some extent reflect the level of photosynthesis (Gururani et al., 2015; Wang et al., 2017). Under salt stress, the chloroplast thylakoid membrane would be destroyed, which could cause a decrease in chlorophyll synthesis and therefore a decrease in chlorophyll concentration (Acosta-Motos et al., 2017; Yang et al., 2020). The chlorophyll concentration remained unchanged in D1 period, but it decreased in the D2 period, implying that short-term droughts had little effect on the photosynthetic rate. As an important osmotic regulator, proline concentration could increase significantly under environmental stress, and it plays important roles in maintaining cellular osmotic pressure, improving antioxidant capacity, storing energy, and detoxifying ammonia (Mansour and Ali, 2017; Wang et al., 2022). Therefore, the proline concentrations in *E. ulmoides* leaves significantly increased under both the salt and drought stress conditions.

### 4.2 Comparisons of non-structural carbohydrates response patterns under drought and salt stress

In this study, SS concentration in leaves, branches and stems decreased, but the ST concentration increased with salt concentration in the S1 period. This may be because salt stress can increase the water column tension in xylem vessels and lead to an increase in cavitation, and therefore plants consume more SS to repair the xylem embolism (Nardini et al., 2011; Tomasella et al., 2020). Meanwhile, past studies showed that an increase in ST can enhance the resistance of plant (Tixier et al., 2018; Guo et al., 2020). The chlorophyll concentration remained unchanged in the D1 period, implying that the photosynthesis rate of *E. ulmoides* was less affected in short-term drought. Therefore, the ST and SS concentrations in leaves remained unchanged, but the concentration of NSCs in branches and stems decreased. The supply of NSCs tends to give priority to the organs most in need (Hartmann et al., 2013). As the most important NSCs source organ, leaves provide the carbon source required for growth and metabolism of trees and should be supplied first. Meanwhile, drought stress hinders the outward transport of photosynthetic products in leaves, resulting in a decrease in stem and branch NSCs (Dietze et al., 2014). Also, the SS concentration in branches decreased, but that in stems remained unchanged, indicating that xylem cavitation might occur in branches and SS was used to repair the embolism (Blum, 2017; Trifilò et al., 2019). The SS concentration in coarse roots and fine roots increased in the S1 period. This is consistent with past studies, implying that plants could distribute more SS to belowground organs to enhance the water uptake during stress (Galvez et al., 2011; Hagedorn et al., 2016). Similarly, fine roots are also important in water absorption under droughts and thus their NSCs is preferentially supplied, leading to significantly higher NSCs concentration in drought treatment groups than that in controls (Hartmann et al., 2013). The ST and NSCs concentrations in coarse roots decreased, while those in fine roots increased, indicating that ST in coarse roots was transformed into SS and transferred to fine roots to maintain their absorption function.

As the salt and drought stress prolonged, the response patterns of each organ showed different variations. In the S2 period, the SS concentration of leaves continued to decrease due to the continuous decrease in chlorophyll concentration and thus the obstruction of photosynthesis. Compare with those in CK, the ST in leaves was converted into SS to maintain their water potential and physiological function and thus leading to an increase of SS concentration in the D2 period. As stress prolonged, the changes of ST and SS concentrations in branches and stems were consistent under two conditions of stress. For details, the SS concentration in branches was low and the ST concentration remained unchanged. This might because branches were prone to embolism, and then SS were used for embolism repair, which leaded to a decrease in the SS concentration (Savi et al., 2016; Blum, 2017). As the degree of the two kinds of stress increased, SS concentration in stems increased, ST concentration decreased, and therefore the total NSCs concentration remained unchanged. This might because higher SS and NSCs concentrations could maintain a higher stem water potential under severe stress conditions and thus prolonging the survival time of plants (O’Brien et al., 2014). Second, as a transport channel, maintaining a high soluble sugar concentration in stem is conducive to maintaining its nutrient transport function and facilitating information transmission between the above- and belowground parts to respond to stress more quickly (Smeekens, 2000; Rolland et al., 2006; Hartmann and Trumbore, 2016).

As drought prolonged, the ST concentration increased and SS concentration decreased in coarse roots. This may be because the increased path resistance and viscosity of phloem sap under stress, as well as the longer distance from source to sink organs, which hindered the transportation of SS from leaves to roots (Sala et al., 2010; Klein et al., 2014). Second, as ST can serve as reserve carbon pools and be used for tissue regeneration and growth, therefore a portion of ST in the stem and fine roots was transferred to the coarse roots to ensure that it could quickly recover in a favorable environment (Trugman et al., 2018; Wiley, 2020). With the prolongation of drought stress, SS in coarse root was transferred to fine root to enhance its water uptake as soil moisture continued to decrease, leading to a decrease in SS in coarse roots. Moreover, ST in fine roots could convert into SS, resulting in an increase in SS and a decrease of ST in fine roots, indicating that fine roots still maintain good water absorption function in short-term drought. Meanwhile, long-term droughts could cause some fine roots to die, and these dead roots would transfer some carbohydrates to living roots before falling off for future tissue regeneration and growth (Martínez-Vilalta et al., 2016; Trugman et al., 2018). In contrast, the concentrations of ST and SS in fine roots decreased under prolonged salt stress. This may be because fine roots firstly responded to salt stress, and their physiological activity was then irreversibly damaged when the osmotic regulation of NSCs is no longer able to offset damages induced by prolonged salt stress (McDowell et al., 2008; Yoshimura et al., 2016). Therefore, plants would transfer SS and ST from fine roots to coarse roots to improve the survival opportunities under stress and aid in the recovery after stress (Wiley, 2020). Meanwhile, the total NSCs concentrations in coarse and fine roots began to decrease as drought prolonged. This indicated that if the drought duration continues to increase, plants can also suffer irreversible damage and may die from carbon starvation in long-term drought. Overall, we compared the similarities and differences of NSCs response patterns in *E. ulmoides* under both salt and drought stress simultaneously, and provided the first evidence that *E. ulmoide*s has contrast NSCs response strategies to these two abiotic stresses.

### 4.3 Relationships between relative growth rate and non-structural carbohydrates concentration and pool size

It was proved that carbon storage could take priority over growth in trees under stress conditions, and thus creating a trade-off between storage and growth (Chantuma et al., 2009; Genet et al., 2010; Wiley and Helliker, 2012). However, we found that RGR showed positive correlations with SS and NSCs concentrations in leaves and branches, which is consistent with the research results of Poorter and Kitajima (2007) and Imaji and Seiwa (2010). This indicated that faster growth was associated with greater carbon remobilization or nutrient stores (Piper, 2020). Myers and Kitajima (2007) found that total NSCs pool size, instead of its concentration, was related to shade tolerance in a neotropical forest. However, RGR was not more sensitive to NSCs pool sizes than concentrations in our study. Moreover, the total SS pool size in leaves was positively related to RGR, but there is a marginal significant negative association between the SS pool size in stems and RGR. This indicated that the relationships between NSCs pool size and growth amongst organs are contrast. The reason for these contrasting results might be that different plants have different priorities for growth and storage under stress conditions: growth-prioritizing plants switch from growth to storage later and completely deplete their carbon storage, while storage-prioritizing plants either do not grow or switch earlier during the stress period (Stefaniak et al., 2024). Overall, the relationship between growth and storage are species-specific, organ dependent, or vary through expressing as concentration or pool size (Genet et al., 2010; Palacio et al., 2014).

## 5 Conclusions

Our study compared the growth, physiology, and NSCs allocation patterns of *E. ulmoides* seedlings under drought and salt stress conditions, which highlights the importance of intensity and duration of stress on NSCs dynamic and thus forest carbon cycling. We found that *E. ulmoides* tended to enhance its stress resistance by increasing leaf thickness or density and decreasing investment in belowground biomass in short-term stress. As salt and drought stress prolonged, LMA maintained at a high level to facilitate drought resistance, and there was a shift in the allocation ratio of above- and belowground biomass. In short-term salt stress, the SS concentration in the leaves, branches, and stems decreased, but the ST concentrations increased to enhance the resistance to stress. However, the ST and SS concentrations in leaves remained unchanged, but total NSCs concentrations in branches and stems decreased in short-term droughts. In both the short-term salt and drought stress, the ST and NSC concentrations in the coarse roots decreased, while the ST and SS concentrations in the fine roots increased to enhance stress resistance and maintain water absorption function. As salt stress prolonged, the physiological activity of fine roots might have suffered irreversible damage, and the ST in fine roots was transferred to coarse roots to improve survival opportunities under stress and assist in the recovery after stress. As drought prolonged, SS in coarse roots were transferred to fine roots and starch in fine roots was also converted into SS to maintain effective water uptake of fine roots, and the total NSCs concentrations in coarse and fine roots began to decrease. This indicated that if the drought duration continues to increase, plants can also suffer irreversible damage and may die from carbon starvation in long-term drought. Significant positive relationships were found between growth and the SS and total NSCs concentrations in leaves and branches, however, no significant correlations were found between growth and below-ground organs. Moreover, relationships between growth and NSCs pool size across organs could be contrast. However, our researches only focused on the morphological and physiological aspects. Further researches should be combined with the -omics technology from a molecular perspective to comprehensively investigate the physiological and carbon allocation mechanisms of trees under environmental changes.

## Data availability statement

The raw data supporting the conclusions of this article will be made available by the authors, without undue reservation.

## Author contributions

XZ: Methodology, Writing – original draft. HQ: Investigation, Software, Writing – original draft. ZK: Investigation, Writing – review & editing. DL: Writing – review & editing. BW: Investigation, Writing – review & editing. SF: Funding acquisition, Methodology, Writing – review & editing. PJ: Funding acquisition, Methodology, Software, Writing – review & editing, Writing – original draft.

## References

[B1] Aaltonen H. Linden A. Heinonsalo J. Biasi C. Pumpanen J. (2017). Effects of prolonged drought stress on Scots pine seedling carbon allocation. Tree Physiol. 37, 418–427. doi: 10.1093/treephys/tpw119 27974653

[B2] Acosta-Motos J. R. Ortuño M. F. Bernal-Vicente A. Diaz-Vivancos P. Sanchez-Blanco M. J. Hernandez J. A. (2017). Plant responses to salt stress: Adaptive mechanisms. Agronomy 7, 18. doi: 10.3390/agronomy7010018

[B3] Adams H. D. Germino M. J. Breshears D. D. Barron-Gafford G. A. Guardiola-Claramonte M. Zou C. B.. (2013). Nonstructural leaf carbohydrate dynamics of Pinus edulis during drought induced tree mortality reveal role for carbon metabolism in mortality mechanism. New Phytol. 197, 1142–1151. doi: 10.1111/nph.12102 23311898

[B4] Anderegg W. R. Berry J. A. Smith D. D. Sperry J. S. Anderegg L. D. Field C. B. (2012). The roles of hydraulic and carbon stress in a widespread climate-induced forest die-off. Proc. Natl. Acad. Sci. U.S.A. 109, 233–237. doi: 10.1073/pnas.1107891109 22167807 PMC3252909

[B5] Aragüés R. Medina E. Zribi W. Clavería I. Alvaro-Fuentes J. Faci J. (2015). Soil salinization as a threat to the sustainability of deficit irrigation under present and expected climate change scenarios. Irrigation Sci. 33, 67–79. doi: 10.1007/s00271-014-0449-x

[B6] Bacher H. Sharaby Y. Walia H. Peleg Z. (2022). Modifying root-to-shoot ratio improves root water influxes in wheat under drought stress. J. Exp. Bot. 73, 1643–1654. doi: 10.1093/jxb/erab500 34791149

[B7] Bartlett M. K. Scoffoni C. Sack L. (2012). The determinants of leaf turgor loss point and prediction of drought tolerance of species and biomes: a global meta-analysis. Ecol. Lett. 15, 393–405. doi: 10.1111/j.1461-0248.2012.01751.x 22435987

[B8] Blum A. (2017). Osmotic adjustment is a prime drought stress adaptive engine in support of plant production. Plant Cell Environ. 40, 4–10. doi: 10.1111/pce.12800 27417527

[B9] Blumstein M. Gersony J. Martínez-Vilalta J. Sala A. (2023). Global variation in nonstructural carbohydrate stores in response to climate. Glob. Change Biol. 29, 1854–1869. doi: 10.1111/gcb.16573 36583374

[B10] Carnicer J. Coll M. Ninyerola M. Pons X. Sánchez G. Peñuelas J. (2011). Widespread crown condition decline, food web disruption, and amplified tree mortality with increased climate change-type drought. Proc. Natl. Acad. Sci. U.S.A. 108, 1474–1478. doi: 10.1073/pnas.1010070108 21220333 PMC3029725

[B11] Chantuma P. Lacointe A. Kasemsap P. Thanisawanyangkura S. Gohet E. Clement A.. (2009). Carbohydrate storage in wood and bark of rubber trees submitted to different level of C demand induced by latex tapping. Tree Physiol. 29, 1021–1031. doi: 10.1093/treephys/tpp043 19556234

[B12] Chapin F. S. Schultze E. Mooney H. (1990). The ecology and economics of storage in plants. Annu. Rev. Ecol. S. 21, 423–447. doi: 10.1146/annurev.ecolsys.21.1.423

[B13] Chaves M. M. Flexas J. Pinheiro C. (2009). Photosynthesis under drought and salt stress: regulation mechanisms from whole plant to cell. Ann. Bot. 103, 551–560. doi: 10.1093/aob/mcn125 18662937 PMC2707345

[B14] Choat B. Brodribb T. J. Brodersen C. R. Duursma R. A. López R. Medlyn B. E. (2018). Triggers of tree mortality under drought. Nature 558, 531–539. doi: 10.1038/s41586-018-0240-x 29950621

[B15] Cimato A. Castelli S. Tattini M. Traversi M. L. (2010). An ecophysiological analysis of salinity tolerance in olive. Environ. Exp. Bot. 68, 214–221. doi: 10.1016/j.envexpbot.2009.12.006

[B16] Cramer W. Guiot J. Fader M. Garrabou J. Gattuso J. P. Iglesias A.. (2018). Climate change and interconnected risks to sustainable development in the Mediterranean. Nat. Clim. Change 8, 972–980. doi: 10.1038/s41558-018-0299-2

[B17] Dickman L. T. McDowell N. G. Grossiord C. Collins A. D. Wolfe B. T. Detto M.. (2019). Homoeostatic maintenance of nonstructural carbohydrates during the 2015–2016 El Niño drought across a tropical forest precipitation gradient. Plant Cell Environ. 42, 1705–1714. doi: 10.1111/pce.13501 30537216

[B18] Dickman L. T. McDowell N. G. Sevanto S. Pangle R. E. Pockman W. T. (2015). Carbohydrate dynamics and mortality in a pinon-juniper woodland under three future precipitation scenarios. Plant Cell Environ. 38, 729–739. doi: 10.1111/pce.12441 25159277

[B19] Dietze M. C. Sala A. Carbone M. S. Czimczik C. I. Mantooth J. A. Richardson A. D.. (2014). Nonstructural carbon in woody plants. Ann. Rev. Plant Biol. 65, 667–687. doi: 10.1146/annurev-arplant-050213-040054 24274032

[B20] Fan L. Dalpé Y. Fang C. Dubé C. Khanizadeh S. (2011). Influence of arbuscular mycorrhizae on biomass and root morphology of selected strawberry cultivars under salt stress. Botany 89, 397–403. doi: 10.1139/b11-028

[B21] Fernández R. J. Wang M. Reynolds J. F. (2002). Do morphological changes mediate plant responses to water stress? A steady-state experiment with two C_4_ grasses. New Phytol. 155, 79–88. doi: 10.1046/j.1469-8137.2002.00438.x 33873299

[B22] Furze M. E. Huggett B. A. Aubrecht D. M. Stolz C. D. Carbone M. S. Richardson A. D. (2019). Whole-tree nonstructural carbohydrate storage and seasonal dynamics in five temperate species. New Phytol. 221, 1466–1477. doi: 10.1111/nph.15462 30368825 PMC6587558

[B23] Galiano L. Martínezvilalta J. Sabaté S. Lloret F. (2012). Determinants of drought effects on crown condition and their relationship with depletion of carbon reserves in a Mediterranean holm oak forest. Tree Physiol. 32, 478–489. doi: 10.1093/treephys/tps025 22499595

[B24] Galvez D. A. Landhaeusser S. M. Tyree M. T. (2011). Root carbon reserve dynamics in aspen seedlings: does simulated drought induce reserve limitation? Tree Physiol. 31, 250–257. doi: 10.1093/treephys/tpr012 21444372

[B25] Garcia-Forner N. Biel C. Savé R. Martínez-Vilalta J. (2017). Isohydric species are not necessarily more carbon limited than anisohydric species during drought. Tree Physiol. 37, 441–455. doi: 10.1093/treephys/tpw109 27885172

[B26] Genet H. Breda N. Dufrene E. (2010). Age-related variation in carbon allocation at tree and stand scales in beech (*Fagus sylvatica* L.) and sessile oak (*Quercus petraea* (Matt.) Liebl.) using a chronosequence approach. Tree Physiol. 30, 177–192. doi: 10.1093/treephys/tpp105 20018984

[B27] Gibon Y. Pyl E.-T. Sulpice R. Lunn J. E. Höhne M. Günther M.. (2009). Adjustment of growth,starch turnover, protein content and central metabolism to a decrease of the carbon supply when Arabidopsis is grown in very short photoperiods. Plant Cell Environ. 32, 859–874. doi: 10.1111/j.1365-3040.2009.01965.x 19236606

[B28] Gruber A. Pirkebner D. Florian C. Oberhuber W. (2012). No evidence for depletion of carbohydrate pools in Scots pine (*Pinus sylvestris* L.) under drought stress. Plant Biol. 14, 142–148. doi: 10.1111/j.1438-8677.2011.00467.x 21974742 PMC3427021

[B29] Guo Q. Wu X. Korpelainen H. Li C. (2020). Stronger intraspecific competition aggravates negative effects of drought on the growth of *Cunninghamia lanceolata.* Environ. Exp. Bot. 175, 104042. doi: 10.1016/j.envexpbot.2020.104042

[B30] Gururani M. A. Venkatesh J. Tran L. S. P. (2015). Regulation of photosynthesis during abiotic stress-induced photoinhibition. Mol. Plant 8, 1304–1320. doi: 10.1016/j.molp.2015.05.005 25997389

[B31] Hagedorn F. Joseph J. Peter M. Luster J. Pritsch K. Geppert U.. (2016). Recovery of trees from drought depends on belowground sink control. Nat. Plants 2, 16111. doi: 10.1038/nplants.2016.111 27428669

[B32] Hartmann H. Trumbore S. (2016). Understanding the roles of nonstructural carbohydrates in forest trees-from what we can measure to what we want to know. New Phytol. 211, 386–403. doi: 10.1111/nph.13955 27061438

[B33] Hartmann H. Ziegler W. Trumbore S. (2013). Lethal drought leads to reduction in nonstructural carbohydrates in Norway spruce tree roots but not in the canopy. Funct. Ecol. 27, 413–427. doi: 10.1111/1365-2435.12046

[B34] He W. Liu H. Qi Y. Liu F. Zhu X. (2020). Patterns in nonstructural carbohydrate contents at the tree organ level in response to drought duration. Global Change Biol. 26, 3627–3638. doi: 10.1111/gcb.15078 32162388

[B35] He X. Wang J. Li M. Hao D. Yang Y. Zhang C.. (2014). *Eucommia ulmoides* Oliv.: Ethnopharmacology, phytochemistry and pharmacology of an important traditional Chinese medicine. J. Ethnopharmacol. 1, 78–92. doi: 10.1016/j.jep.2013.11.023 24296089

[B36] Hoch G. Richter A. Körner C. (2003). Non-structural carbon compounds in temperate forest trees. Plant Cell. Environ. 26, 1067–1081. doi: 10.1046/j.0016-8025.2003.01032.x

[B37] Iglesias D. J. Lliso I. Tadeo F. R. Talon M. (2002). Regulation of photosynthesis through source:sink imbalance in citrus is mediated by carbohydrate content in leaves. Physiol. Plant 116, 563–572. doi: 10.1034/j.1399-3054.2002.1160416.x

[B38] Imaji A. Seiwa K. (2010). Carbon allocation to defense, storage, and growth in seedlings of two temperate broad-leaved tree species. Oecologia 162 (2), 273–281. doi: 10.1007/s00442-009-1453-3 19763628

[B39] Jacqueline S. Kay M. Paul C. E. Boon P. I. (2006). Interactive effects of salinity and water depth on the growth of *Melaleuca ericifolia* Sm. (Swamp paperbark) seedlings. Aquat. Bot. 86 (3), 213–222. doi: 10.1016/j.aquabot.2006.10.002

[B40] Jiao L. Zhou Y. Liu X. Wang S. Li F. (2020). Driving forces analysis of non-structural carbohydrates for *Phragmites australis* in different habitats of inland river wetland. Water 12, 1700. doi: 10.3390/w12061700

[B41] John G. P. Scoffoni C. Buckley T. N. Villar R. Poorter H. Sack L. (2017). The anatomical and compositional basis of leaf mass per area. Ecol. Lett. 20, 412–425. doi: 10.1111/ele.12739 28198076

[B42] Joslin J. Wolfe M. Hanson P. (2000). Effects of altered water regimes on forest root systems. New Phytol. 147, 117–129. doi: 10.1046/j.1469-8137.2000.00692.x

[B43] Kanai M. Higuchi K. Hagihara T. Konishi T. Ishii T. Fujita N.. (2007). Common reed produces starch granules at the shoot base in response to salt stress. New Phytol. 176, 572–580. doi: 10.1111/j.1469-8137.2007.02188.x 17953542

[B44] Kannenberg S. A. Novick K. A. Phillips R. P. (2018). Coarse roots prevent declines in whole-tree non-structural carbohydrate pools during drought in an isohydric and an anisohydric species. Tree Physiol. 38, 582–590. doi: 10.1093/treephys/tpx119 29036648

[B45] Kir L. P. Weisbach A. N. Weiner J. (2013). Root and shoot competition: a meta-analysis. J. Ecol. 101, 1298–1312. doi: 10.1111/1365-2745.12129

[B46] Klein T. Hoch G. Yakir D. Körner C. (2014). Drought stress, growth and nonstructural carbohydrate dynamics of pine trees in a semi-arid forest. Tree Physiol. 34, 981–992. doi: 10.1093/treephys/tpu071 25187568

[B47] Latt C. R. Nair P. K. R. Kang B. T. (2001). Reserve carbohydrate levels in the boles and structural roots of five multipurpose tree species in a seasonally dry tropical climate. For. Ecol. Manage. 146, 146–158. doi: 10.1016/S0378-1127(00)00456-4

[B48] Li L. Liu M. Shi K. Yu Z. Zhou Y. Fan R.. (2019). Dynamic changes in metabolite accumulation and the transcriptome during leaf growth and development in *Eucommia ulmoides* . Int. J. Mol. Sci. 20, 4030. doi: 10.3390/ijms20164030 31426587 PMC6721751

[B49] Li W. Hartmann H. Adams H. D. Zhang H. Jin C. Zhao C.. (2018). The sweet side of global change–dynamic responses of non-structural carbohydrates to drought, elevated CO_2_ and nitrogen fertilization in tree species. Tree Physiol. 38, 1706–1723. doi: 10.1093/treephys/tpy059 29897549

[B50] Li Y. Duan B. L. Chen J. Korpelainen H. Niinemets Ü. Li C. (2016). Males exhibit competitive advantages over females of Populus deltoides under salinity stress. Tree Physiol. 36, 1573–1584. doi: 10.1093/treephys/tpw070 27587482

[B51] Li Y. Tian D. Yang H. Niu S. (2018). Size-dependent nutrient limitation of tree growth from subtropical to cold temperate forests. Funct. Ecol. 32, 95–105. doi: 10.1111/1365-2435.12975

[B52] Liu W. S. Zheng L. Qi D. H. (2020). Variation in leaf traits at different altitudes reflects the adaptive strategy of plants to environmental changes. Ecol. Evol. 10, 166–8175. doi: 10.1002/ece3.6519 PMC7417217 32788969

[B53] Liu X. Wang X. Z. Kang K. Sun G. T. Zhu M. Q. (2022). Review on extraction, characteristic, and engineering of the *Eucommia ulmodies* rubber for industrial application. Ind. Crop Prod. 180, 114733. doi: 10.1016/j.indcrop.2022.114733

[B54] Luo Z. Li K. Gai Y. Göbel C. Wildhagen H. Jiang X.. (2011). The ectomycorrhizal fungus (*Paxillus involutus*) modulates leaf physiology of poplar towards improved salt tolerance. Environ. Exp. Bot. 72, 304–311. doi: 10.1016/j.envexpbot.2011.04.008

[B55] Mansour M. M. F. Ali E. F. (2017). Evaluation of proline functions in saline conditions. Phytochemistry 140, 52–68. doi: 10.1016/j.phytochem.2017.04.016 28458142

[B56] Martínez-Vilalta J. Sala A. Asensio D. Galiano L. Hoch G. Palacio S.. (2016). Dynamics of non-structural carbohydrates in terrestrial plants: a global synthesis. Ecol. Monogr. 86, 495–516. doi: 10.1002/ecm.1231

[B57] McDowell N. G. (2011). Mechanisms linking drought, hydraulics, carbon metabolism, and vegetation mortality. Plant Physiol. 155, 1051–1059. doi: 10.1104/pp.110.170704 21239620 PMC3046567

[B58] McDowell N. G. Allen C. D. Anderson-Teixeira K. Aukema B. H. Bond-Lamberty B. Chini L.. (2020). Pervasive shifts in forest dynamics in a changing world. Science 368, eaaz9463. doi: 10.1126/science.aaz9463 32467364

[B59] McDowell N. G. Pockman W. T. Allen C. D. Breshears D. D. Cobb N. Kolb T.. (2008). Mechanisms of plant survival and mortality during drought: why do some plants survive while others succumb to drought? New Phytol. 178, 719–739. doi: 10.1111/j.1469-8137.2008.02436.x 18422905

[B60] Mitchell P. J. Grady A. P. O. Tissue D. T. White D. A. Ottenschlaeger M. L. Pinkard E. A. (2013). Drought response strategies define the relative contributions of hydraulic dysfunction and carbohydrate depletion during tree mortality. New Phytol. 197, 862–872. doi: 10.1111/nph.12064 23228042

[B61] Moser B. Kipfer T. Richter S. Egli S. Wohlgemuth T. (2015). Drought resistance of *Pinus sylvestris* seedlings conferred by plastic root architecture rather than ectomycorrhizal colonization. Ann. For. Sci. 72, 303–309. doi: 10.1007/s13595-014-0380-6

[B62] Munns R. (2002). Comparative physiology of salt and water stress. Plant Cell Environ. 25, 239–250. doi: 10.1046/j.0016-8025.2001.00808.x 11841667

[B63] Myers J. A. Kitajima K. (2007). Carbohydrate storage enhances seedling shade and stress tolerance in a neotropical forest. J. Ecol. 95, 383–395. doi: 10.1111/j.1365-2745.2006.01207.x

[B64] Nardini A. Lo Gullo M. A. Salleo S. (2011). Refilling embolized xylem conduits: Is it a matter of phloem unloading? Plant Sci. 180, 604–611. doi: 10.1016/j.plantsci.2010.12.011 21421408

[B65] Nordborg F. Welander N. T. (2001). Growth responses of rooted cuttings from five clones of *Picea abies* (L.) Karst. after a short drought period. Scand. J. For. Res. 16, 324–330. doi: 10.1080/713785145

[B66] O’Brien M. J. Leuzinger S. Philipson C. D. Tay J. Hector A. (2014). Drought survival of tropical tree seedlings enhanced by non-structural carbohydrate levels. Nat. Clim. Change 4, 710–714. doi: 10.1038/nclimate2281

[B67] Palacio S. Hoch G. Sala A. Körner C. Millard P. (2014). Does carbon storage limit tree growth? New Phytol. 201, 1096–1100. doi: 10.1111/nph.12602 24172023

[B68] Piper F. I. (2020). Decoupling between growth rate and storage remobilization in broadleaf temperate tree species. Funct. Ecol. 34, 1180–1192. doi: 10.1111/1365-2435.13552

[B69] Piper F. I. Fajardo A. Hoch G. (2017). Single-provenance mature conifers show higher non-structural carbohydrate storage and reduced growth in a drier location. Tree Physiol. 37, 1001–1010. doi: 10.1093/treephys/tpx061 28549182

[B70] Piper F. I. Moreno-Meynard P. Fajardo A. (2022). Nonstructural carbohydrates predict survival in saplings of temperate trees under carbon stress. Funct. Ecol. 36, 2806–2818. doi: 10.1111/1365-2435.14158

[B71] Poorter L. Kitajima K. (2007). Carbohydrate storage and light requirements of tropical moist and dry forest tree species. Ecology 88 (4), 1000–1011. doi: 10.1890/06-0984 17536715

[B72] Polle A. Chen S. (2015). On the salty side of life: molecular, physiological and anatomical adaptation and acclimation of trees to extreme habitats. Plant Cell Environ. 38, 1794–1816. doi: 10.1111/pce.12440 25159181

[B73] Rolland F. Baena-Gonzalez E. Sheen J. (2006). Sugar sensing and signaling in plants: conserved and novel mechanisms. Ann. Rev. Plant Biol. 57, 675–709. doi: 10.1146/annurev.arplant.57.032905.105441 16669778

[B74] Rosas T. Galiano L. Ogaya R. Penuelas J. Martinez-Vilalta J. (2013). Dynamics of non-structural carbohydrates in three Mediterranean woody species following long-term experimental drought. Front. Plant Sci. 4. doi: 10.3389/fpls.2013.00400 PMC3795346 24130568

[B75] Sala A. Piper F. Hoch G. (2010). Physiological mechanisms of drought-induced tree mortality are far from being resolved. New Phytol. 186, 274–281. doi: 10.1111/j.1469-8137.2009.03167.x 20409184

[B76] Sala A. Woodruff D. R. Meinzer F. C. (2012). Carbon dynamics in trees: feast or famine? Tree Physiol. 32, 764–775. doi: 10.1093/treephys/tpr143 22302370

[B77] Salter J. Morris K. Bailey P. C. E. Boon P. I. (2006). Interactive effects of salinity and water depth on the growth of *Melaleuca ericifolia* Sm. (Swamp paperbark) seedlings. Aquat. Bot. 86, 213–222. doi: 10.1016/j.aquabot.2006.10.002

[B78] Sancho-Knapik D. Escudero A. Mediavilla S. Scoffoni C. Zailaa J. Cavender-Bares J.. (2021). Deciduous and evergreen oaks show contrasting adaptive responses in leaf mass per area across environments. New Phytol. 230, 521–534. doi: 10.1111/nph.17151 33340114

[B79] Sapes G. Demaree P. Lekberg Y. Sala A. (2021). Plant carbohydrate depletion impairs water relations and spreads *via* ectomycorrhizal networks. New Phytol. 229, 3172–3183. doi: 10.1111/nph.17134 33280134

[B80] Savi T. Casolo V. Luglio J. Bertuzzi S. Trifilo’ P. Lo Gullo M. A.. (2016). Species-specific reversal of stem xylem embolism after a prolonged drought correlates to endpoint concentration of soluble sugars. Plant Physiol. Biochem. 106, 198–207. doi: 10.1016/j.plaphy.2016.04.051 27174138

[B81] Secchi F. Zwieniecki M. A. (2011). Sensing embolism in xylem vessels: the role of sucrose as a trigger for refilling. Plant Cell. Environ. 34, 514–524. doi: 10.1111/j.1365-3040.2010.02259 21118423

[B82] Silpi U. Lacointe A. Kasempsap P. Thanysawanyangkura S. Chantuma P. Gohet E.. (2007). Carbohydrate reserves as a competing sink: evidence from tapping rubber trees. Tree Physiol. 27, 881–889. doi: 10.1093/treephys/27.6.881 17331906

[B83] Smeekens S. (2000). Sugar-induced signal transduction in plants. Ann. Rev. Plant Biol. 51, 49–81. doi: 10.1146/annurev.arplant.51.1.49 15012186

[B84] Stefaniak E. Z. Tissue D. T. Dewar R. C. Medlyn B. E. (2024). Optimal carbon storage during drought. Tree Physiol. doi: 10.1093/treephys/tpae032 PMC11898659 38498322

[B85] Tixier A. Orozco J. Roxas A. A. Earles J. M. Zwieniecki M. A. (2018). Diurnal variation in nonstructural carbohydrate storage in trees: remobilization and vertical mixing. Plant Physiol. 178, 1602–1613. doi: 10.1104/pp.18.00923 30366979 PMC6288742

[B86] Tomasella M. Petrussa E. Petruzzellis F. Nardini A. Casolo V. (2020). The possible role of non-structural carbohydrates in the regulation of tree hydraulics. Int. J. Mol. Sci. 21, 144. doi: 10.3390/ijms21010144 PMC6981889 31878253

[B87] Trenberth K. E. Dai A. van der Schrier G. Jones P. D. Barichivich J. Briffa K. R.. (2014). Global warming and changes in drought. Nat. Clim. Change 4, 17–22. doi: 10.1038/nclimate2067

[B88] Trifilò P. Kiorapostolou N. Petruzzellis F. Vitti S. Petit G. Lo Gullo M. A.. (2019). Hydraulic recovery from xylem embolism in excised branches of twelve woody species: Relationships with parenchyma cells and non-structural carbohydrates. Plant Physiol. Biochem. 139, 513–520. doi: 10.1016/j.plaphy.2019.04.013 31015090

[B89] Trugman A. T. Detto M. Bartlett M. K. Medvigy D. Anderegg W. R. L. Schwalm C.. (2018). Tree carbon allocation explains forest drought-kill and recovery patterns. Ecol. Lett. 21, 1552–1560. doi: 10.1111/ele.13136 30125446

[B90] Wang B. Zhang J. Pei D. Yu L. (2021). Combined effects of water stress and salinity on growth, physiological, and biochemical traits in two walnut genotypes. Physiol. Plantarum 172, 176–187. doi: 10.1111/ppl.13316 33314146

[B91] Wang L. Du H. Li T. Wuyun T. N. (2018). *De novo* transcriptome sequencing and identification of genes Related to salt stress in *Eucommia ulmoides* Oliver. Trees-Struct. Funct. 32, 151–163. doi: 10.1007/s00468-017-1620-9

[B92] Wang T. Yang W. Xie Y. Shi D. W. Ma Y. L. Sun X. (2017). Effects of exogenous nitric oxide on the photosynthetic characteristics of bamboo (*Indocalamus barbatus* McClure) seedlings under acid rain stress. Plant Growth Regul. 82, 69–78. doi: 10.1007/s10725-016-0239-y

[B93] Wang Z. Yang Y. Yadav V. Zhao W. He Y. Zhang X.. (2022). Drought-induced proline is mainly synthesized in leaves and to roots in watermelon under water deficit. Hortic. Plant J. 8, 15–626. doi: 10.1016/j.hpj.2022.06.009

[B94] Wiley E. (2020). Do carbon reserves increase tree survival during stress and following disturbance? Curr. For. Rep. 6, 14–25. doi: 10.1007/s40725-019-00106-2

[B95] Wiley E. Helliker B. (2012). A re-evaluation of carbon storage in trees lends greater support for carbon limitation to growth. New Phytol. 195, 285–289. doi: 10.1111/j.1469-8137.2012.04180.x 22568553

[B96] Xu Z. Wang J. Zhen W. Sun T. Hu X. (2022). Abscisic acid alleviates harmful effect of saline-alkaline stress on tomato seedlings. Plant Physiol. Biochem. 175, 58–67. doi: 10.1016/j.plaphy.2022.01.018 35180529

[B97] Yang C. Lin C. Kao C. (1999). Endogenous ornithine and arginine contents and dark-induced proline accumulation in detached rice leaves. J. Plant Physiol. 155, 665–668. doi: 10.1016/S0176-1617(99)80080-7

[B98] Yang H. Dai L. J. Wei Y. X. Deng Z. Li D. J. (2020). Melatonin enhances salt stress tolerance in rubber tree (*Hevea brasiliensis*) seedlings. Ind. Crop Prod. 145, 111990. doi: 10.1016/j.indcrop.2019.111990

[B99] Yoshimura K. Saiki S.-T. Yazaki K. Ogasa M. Y. Shirai M. Nakano T.. (2016). The dynamics of carbon stored in xylem sapwood to drought-induced hydraulic stress in mature trees. Sci. Rep. 6, 24513. doi: 10.1038/srep24513 27079677 PMC4832204

[B100] Yu L. Tang S. Guo C. Korpelainen H. Li C. (2023). Differences in ecophysiological responses of *Populus euphratica* females and males exposed to salinity and alkali stress. Plant Physiol. Biochem. 198, 107707. doi: 10.1016/j.plaphy.2023.107707 37086693

[B101] Zhang J. Flowers T. J. Wang S. (2010). Mechanisms of sodium uptake by roots of higher plants. Plant Soil 326, 45–60. doi: 10.1007/s11104-009-0076-0

[B102] Zhang P. Zhou X. Fu Y. Shao J. Zhou L. Li S.. (2020). Differential effects of drought on nonstructural carbohydrate storage in seedlings and mature trees of four species in a subtropical forest. For. Ecol. Manage. 469, 118159. doi: 10.1016/j.foreco.2020.118159

[B103] Zhao H. Yao X. Lu L. (2023). The amplification and activity analysis of the a small rubber particle protein (SRPP) promoter of *Eucommia ulmoides* (EuSRPP) revealed that its activity was regulated by MeJA, GA3, and drought pathways. Horticulturae 9, 856. doi: 10.3390/horticulturae9080856

[B104] Zhou Q. Shi H. He R. Liu H. Zhu W. Yu D.. (2021). Prioritized carbon allocation to storage of different functional types of species at the upper range limits is driven by different environmental drivers. Sci. Total Environ. 773, 145581. doi: 10.1016/j.scitotenv.2021.145581 33582346

[B105] Zhu M. Sun R. (2018). *Eucommia ulmoides* Oliver: A potential feedstock for bioactive products. J. Agric. Food Chem. 66, 5433–5438. doi: 10.1021/acs.jafc.8b01312 29745662

[B106] Zhu S. Nong J. Luo G. Li Q. Wang F. Jiang D.. (2021). Varied tolerance and different responses of five citrus rootstocks to acid stress by principle component analysis and orthogonal analysis. Sci. Hortic. 278, 109853. doi: 10.1016/j.scienta.2020.109853

[B107] Zuo Y. Li B. Guan S. Jia J. Xu X. Zhang Z.. (2022). EuRBG10 involved in indole alkaloids biosynthesis in *Eucommia ulmoides* induced by drought and salt stresses. J. Plant Physiol. 278, 153813. doi: 10.1016/j.jplph.2022.153813 36179396

