# Supplementary material for: Growth and non-structural carbohydrates response patterns of Eucommia ulmoides under salt and drought stress
Source: Front Plant Sci. 2024 Jul 18;15:1436152. doi: 10.3389/fpls.2024.1436152 (PMC11291362; doi:10.3389/fpls.2024.1436152)
Supplement: Supplementary file 1 [file DataSheet_1.docx]

Supplementary Material

**Supplementary Table 1.** Relationships between relative growth rate (RGR) and SS/ST/NSCs concentration in below-ground organ

| Compositions | *R*^2^ | *P* |
| --- | --- | --- |
| SS concentration in coarse root | 0.088 | 0.350 |
| ST concentration in coarse root | 0.081 | 0.371 |
| Total NSC concentration in coarse root | 0.045 | 0.509 |
| SS concentration in fine root | 0.114 | 0.282 |
| ST concentration in fine root | 0.001 | 0.935 |
| Total NSC concentration in fine root | 0.051 | 0.480 |

**Supplementary Table 2.** Relationships between relative growth rate (RGR) and SS/ST/NSCs pool size in below-ground organ and the whole plant level

| Compositions | *R*^2^ | *P* |
| --- | --- | --- |
| SS pool in coarse root | 0.084 | 0.360 |
| ST pool in coarse root | 0.006 | 0.813 |
| Total NSC pool in coarse root | 0.000 | 0.977 |
| SS pool in fine root | 0.000 | 0.963 |
| ST pool in fine root | 0.041 | 0.527 |
| Total NSC pool in fine root | 0.034 | 0.569 |
| SS pool at whole plant level | 0.058 | 0.450 |
| ST pool at whole plant level | 0.006 | 0.817 |
| Total NSC pool at whole plant level | 0.012 | 0.738 |


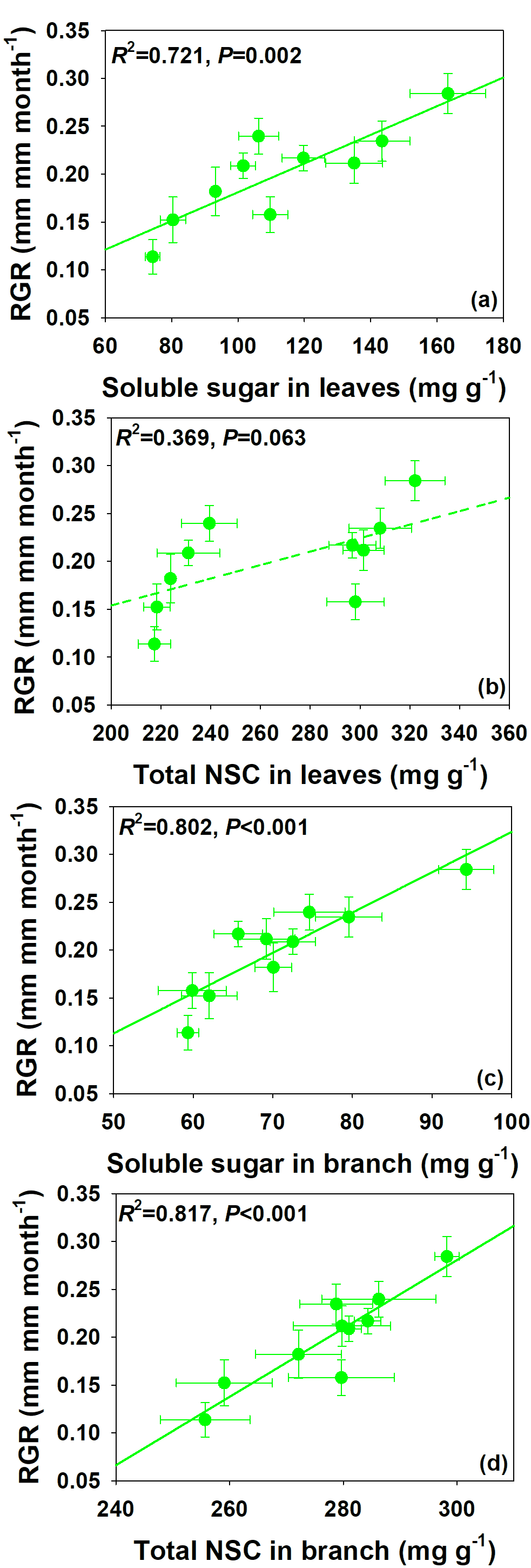


**Supplementary Figure 1.** Relationships between relative growth rate (RGR) and soluble sugar and total NSCs concentrations in leaves (a, b) and branch (c, d) under salt stress.


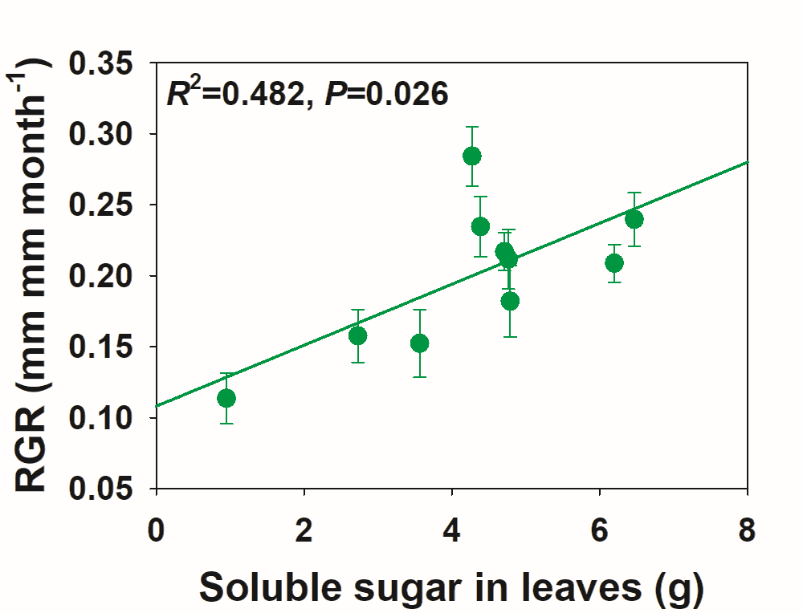


**Supplementary Figure 2.** Relationship between relative growth rate (RGR) and soluble sugar pool size in leaves under salt stress.
